# Revision of Unicompartmental Knee Arthroplasty to Total Knee Arthroplasty Using the CORI^TM^ Imageless System: A Step-By-Step Surgical Technique

**DOI:** 10.1051/sicotj/2026045

**Published:** 2026-10-09

**Authors:** Dan Croitor, Régis Pailhé, Alexandre Le Guen, Ali Alayane, Étienne Cavaignac, Alessandro Carrozzo

**Affiliations:** 1 Department of Orthopaedic Surgery, Hôpital Pierre Paul Riquet, CHU de Toulouse 1 Place du Docteur Baylac 31000 Toulouse France; 2 University for Continuing Education Krems Dr.-Karl-Dorrek-Strasse 30 3500 Krems Austria; 3 Clinique Aguilera Ramsay Santé Biarritz 6 rue de l’Estagnas 64200 Biarritz France; 4 Department of Orthopaedic Surgery, Institut Mutualiste Montsouris (IMM) 42 Bd Jourdan 75014 Paris France; 5 Università degli Studi “Link Campus University”, Dipartimento di Scienze della Vita, della Salute e delle Professioni Sanitarie Via del Casale di S. Pio V 44 00165 Rome Italy

**Keywords:** Imageless robotic-assisted total knee arthroplasty, Unicompartmental knee arthroplasty revision, CORI system, Aseptic loosening, Conversion surgery

## Abstract

Conversion of unicompartmental knee arthroplasty (UKA) to total knee arthroplasty (TKA) is technically demanding due to altered anatomy, asymmetric bone loss, and soft-tissue imbalance, often necessitating stems or augments. Robotic-assisted TKA may enhance the accuracy of bone resection and ligament balancing, potentially facilitating bone preservation in this complex setting. This article describes a step-by-step surgical technique for imageless robotic-assisted conversion of a failed medial UKA to TKA using the CORI™ system in a case of early aseptic loosening. The workflow is based on intraoperative surface mapping and dynamic kinematic assessment. The knee is initially registered as a native joint to capture global alignment and soft-tissue behavior, followed by additional reference point acquisition after implant removal to accurately quantify bone loss. This strategy enables patient-specific planning of femoral and tibial resections, real-time adjustment of component positioning, and controlled gap balancing throughout the range of motion. The principal innovation of this technique is the dual-stage imageless robotic workflow, combining pre-explant native kinematic registration with post-explant defect mapping and iterative re-planning. In the presented case, conversion was successfully achieved without the use of stems or augments. Imageless robotic-assisted UKA-to-TKA conversion using this technique appears feasible and reproducible. By combining native kinematic mapping with quantitative defect assessment, this workflow may facilitate accurate implant positioning, controlled gap balancing, and bone-preserving reconstruction in selected cases.

## Introduction

Conversion of unicompartmental knee arthroplasty (UKA) to total knee arthroplasty (TKA) remains technically demanding. Implant removal, asymmetric bone loss, altered joint line anatomy, and soft-tissue imbalance frequently necessitate stems or augments, increasing surgical complexity and reducing bone preservation [1].

In cases of aseptic loosening, mechanical bone loss may further compromise residual bone stock, making accurate resection planning challenging. Conventional techniques rely on visual estimation and standard mechanical guides, often in the absence of reliable anatomical landmarks. Thus, in patients with aseptic loosening, robotic-assisted conversion of UKA to TKA may be useful [2].

Robotic-assisted TKA has demonstrated improved accuracy in bone resection and ligament balancing. Unlike primary TKA procedures, which often allow for more straightforward preoperative planning and intraoperative flow, revision surgery frequently necessitates real-time modifications based on the condition of the bone loss and surrounding soft tissues encountered during the operation [3].

The workflow of imageless robotic systems during UKA-to-TKA conversion has previously been described in the literature [4]. However, the principal novelty of the present report lies in the use of a dual-stage imageless robotic workflow with the CORI™ system. The knee is first registered as a native joint prior to implant removal, allowing capture of global alignment and soft-tissue kinematics before surgical alteration of the joint. Following the explantation, additional robotic reference points are acquired to quantify bone defects and update the three-dimensional model. This iterative strategy enables real-time refinement of resection depth, component positioning, and gap balancing, potentially facilitating bone-preserving conversion without the routine use of stems or augments.

The objective of this article is to describe this step-by-step imageless robotic workflow using the CORI™ system and to illustrate how native kinematic registration combined with post-explant defect quantification may assist UKA-to-TKA conversion.

## Surgical technique

The complete surgical workflow is available as Supplementary Video 1. Figure 1 illustrates preoperative X-rays.

**Figure 1:**
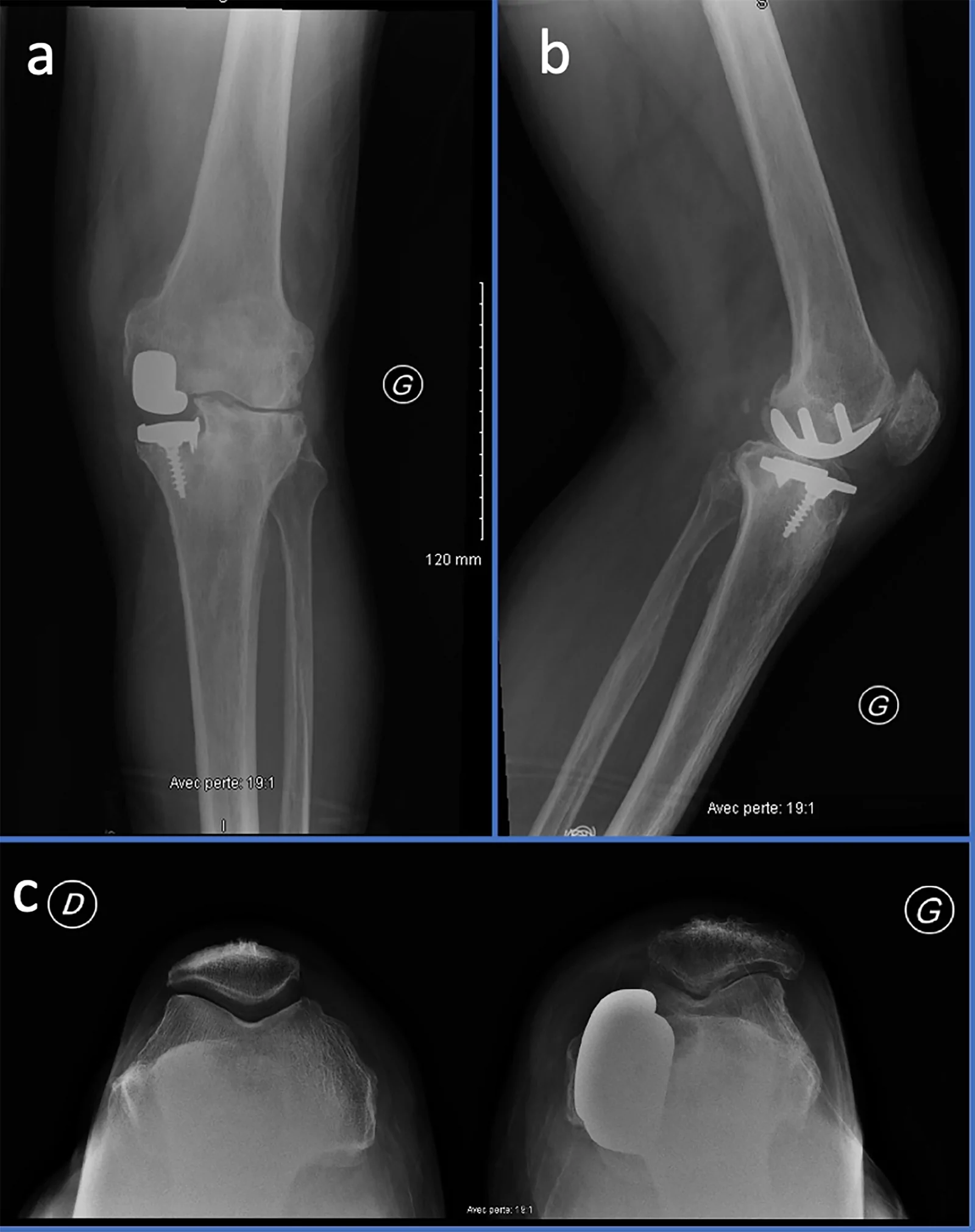
Preoperative radiographs of the left knee demonstrate a mobilized medial unicompartmental knee arthroplasty: (a) anteroposterior view; (b) lateral view; and (c) axial (patellofemoral) view.

Technical Pearls & Pitfalls in Imageless Robotic UKA-to-TKA Conversion are presented below in Table 1.

*Step 1 – Surgical approach*: The revision procedure was performed using the CORI™ (Smith & Nephew) imageless robotic-assisted platform under general anesthesia and tourniquet-free. The patient was placed supine, with a lateral and distal pad maintaining 90° knee flexion.
A standard subvastus approach was utilized. The previous implant was carefully exposed, and soft tissues were protected.
*Step 2 – Registration and planning*: Anatomic landmarks were identified, including the medial and lateral malleoli and the centers of the tibia and femur. Hip center acquisition was obtained by passive circumduction, followed by kinematic registration through a full range of motion. The knee was treated as a native joint during acquisition. Femoral and tibial surface mapping was completed to generate a three-dimensional model, with missing points automatically highlighted by the system (Figure 2).
*Step 3 – Ligament balancing and pre-resection planning*: The CORI system applies a restricted kinematic alignment strategy, supplemented by dynamic functional balancing based on intraoperative soft-tissue assessment (Figure 3).
*Step 4 – Implant removal and bone loss assessment*: The failed components were removed. The CORI™ system allowed acquisition of additional reference points on both the femur and tibia to quantify bone loss. In this case, the femoral and tibial defects corresponded to AORI Type 1, indicating minor contained bone loss without significant metaphyseal deficiency.
These points were visualized in pink on the robotic display, precisely delineating defect areas. Computer mapping of the residual femoral bone surface after implant removal is a helpful guide in minimizing resection depth.
*Step 5 – Planning*: Adjustments were made using robotic planning software to balance the gaps (Figure 4).
In extension, valgus correction and minor additional tibial resection were planned to optimize coronal alignment while maintaining the principles of restricted kinematic alignment. Flexion adjustments were fine-tuned to avoid femoral notching, achieving symmetrical gaps in both planes. Implant positioning was iteratively refined according to intraoperative gap measurements and kinematic evaluation to achieve balanced flexion–extension spaces and stable knee mechanics throughout the range of motion.
*Step 6 – Bone preparation*: Once resection planning was validated, robotic-guided resections were performed. The femoral and tibial cuts were executed using a burr, and alignment accuracy was confirmed before resection. Bone preparation preserved the host bone, and all remnants of the previous implant were removed (Figure 5).
*Step 7 – Trialing, final implantation and wound closure*: Trial components were placed to confirm alignment and ligament balance. In this case, an 11 mm polyethylene insert was selected based on intraoperative gap balancing to restore joint line height and achieve symmetric flexion–extension stability following implant removal. This implant size was not used as a standard recommendation for UKA-to-TKA conversion, but rather individualized according to soft-tissue tension and defect-related changes in joint space.

**Table 1:** Technical Pearls & Pitfalls in imageless robotic UKA-to-TKA conversion.

Pearls	Pitfalls
Perform native registration before implant removal	Inaccurate surface mapping if implant interferes
Capture hip center prior to explantation	Registration drift after explantation
Acquire supplementary reference points after component removal to quantify defects	Overcorrection during valgus adjustment
Iterative planning after defect quantification	Failure to reassess gaps after tibial recut
Preserve bone stock through conservative resections	

**Figure 2:**
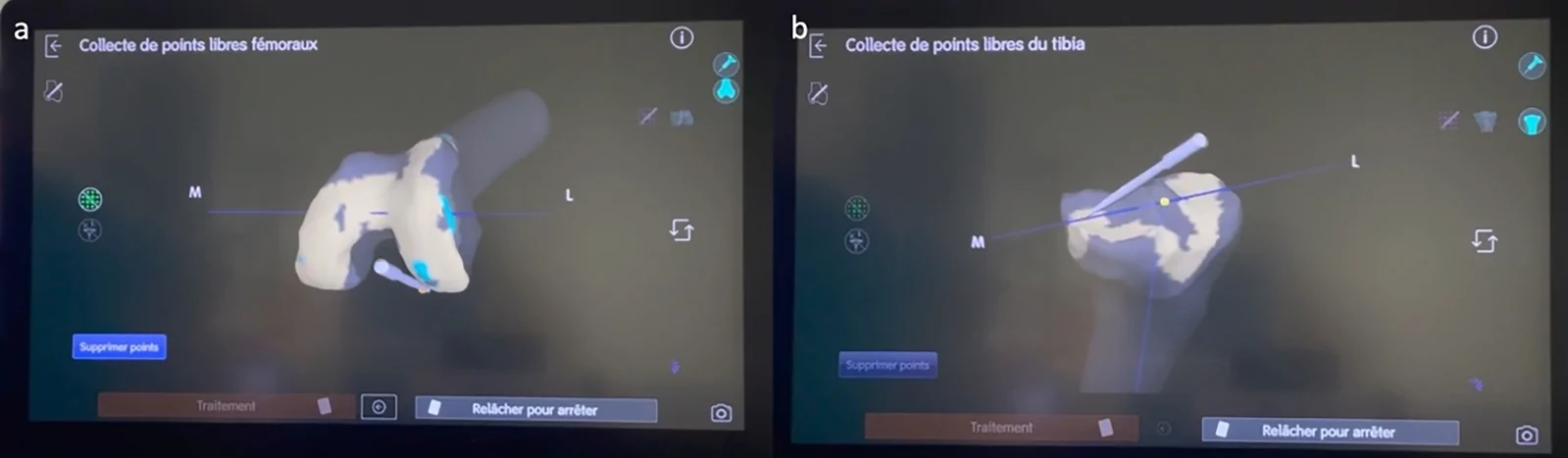
Intraoperative screenshots of imageless robotic surface mapping prior to implant removal. (a) Acquisition of femoral reference points during surface mapping; (b) acquisition of tibial reference points. The knee is initially treated as a native joint to enable kinematic registration and three-dimensional reconstruction before component explantation.

**Figure 3:**
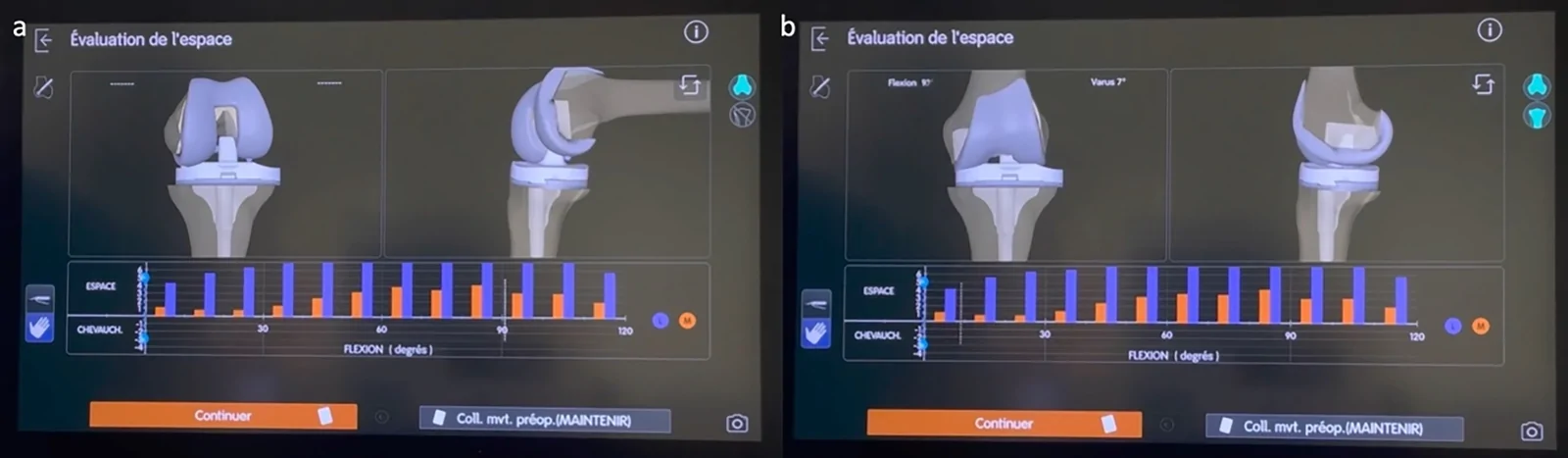
Intraoperative ligament assessment using the imageless robotic platform. (a) Evaluation of coronal balance in full extension; (b) assessment at full flexion. The lower graph displays dynamic gap measurements throughout the entire range of motion, allowing real-time analysis of medial and lateral compartment balance across flexion.

**Figure 4:**
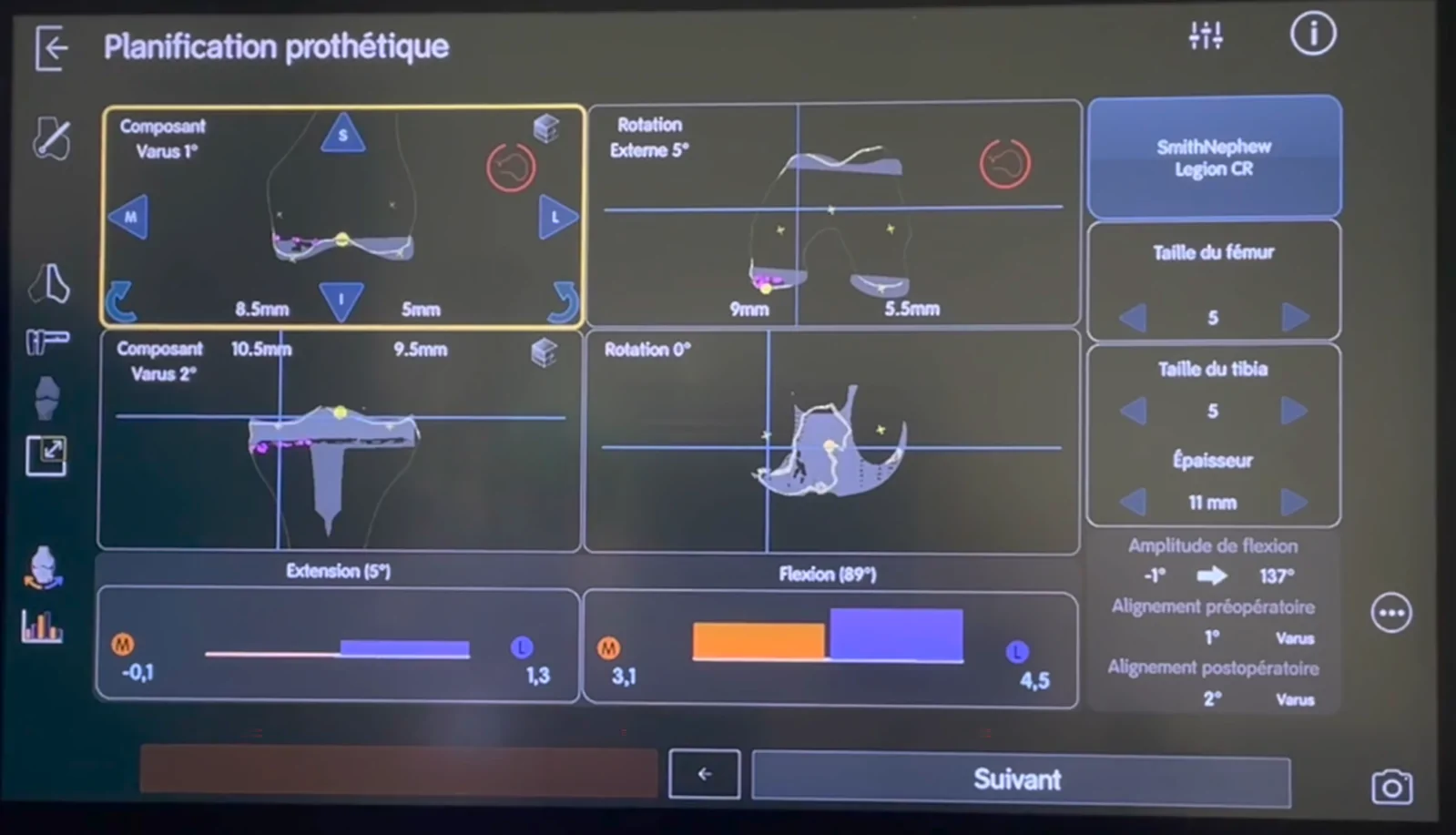
Intraoperative prosthetic planning using the imageless robotic platform. The interface displays component positioning parameters, including femoral and tibial alignment in the coronal and axial planes, planned varus–valgus correction, rotational alignment, resection depth, implant size selection, and predicted flexion–extension gap balance across the range of motion.

**Figure 5:**
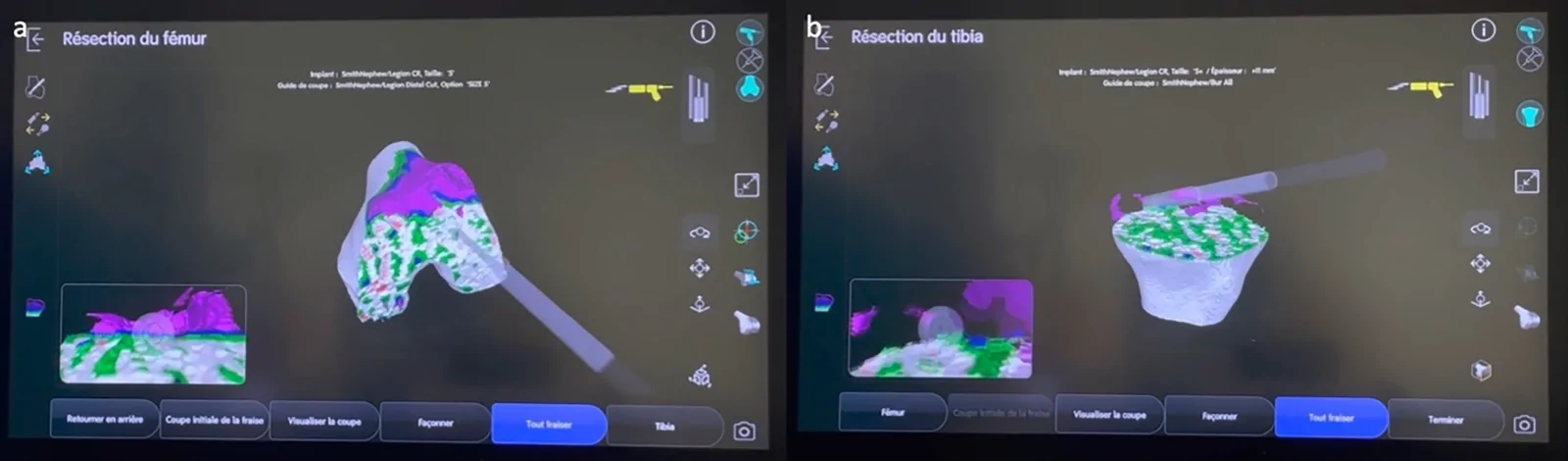
Robotic-guided bone resections during UKA-to-TKA conversion. (a) Femoral resection performed according to the preoperative plan using real-time robotic guidance; (b) tibial resection executed under robotic control.

Full range of motion testing demonstrated stability and smooth kinematics. The final implants, either CS or PS fixed bearing designs, are positioned. In the presented case, a Legion TKS implant (Smith and Nephew) was used. Final implantation was performed using a cemented technique: tibial baseplate, polyethylene insert, femoral component.

The robotic-assisted conversion was completed without complications, and the entire procedure was performed without stems or augments, and bone preservation was optimized.

Postoperatively, the patient achieved early full range of motion and significant pain reduction. No wound complications, instability, or fractures were observed. Early radiographs demonstrated correct component alignment and stable fixation (Figure 6).

**Figure 6:**
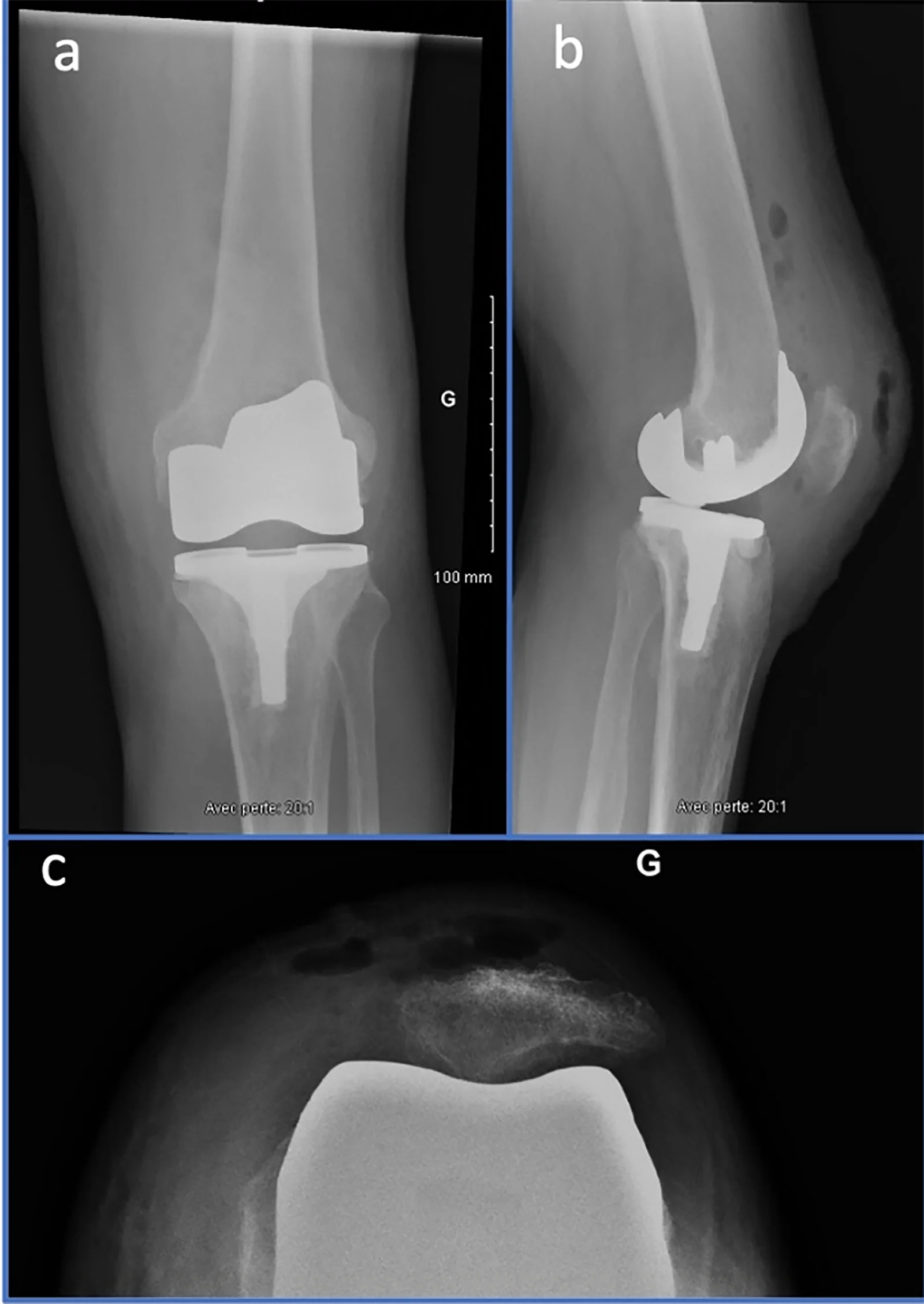
Postoperative radiographs of the left knee after converting the medial unicompartmental knee arthroplasty to total knee replacement (Legion, Smith and Nephew): (a) anteroposterior view; (b) lateral view; and (c) axial (patellofemoral) view.

## Discussion

Conversion of a failed unicompartmental knee arthroplasty (UKA) to total knee arthroplasty (TKA) remains technically demanding due to asymmetric bone loss, altered joint lines, and the frequent need for stems or augments [1].

Conventional conversion techniques rely on visual estimation and mechanical alignment guides, often in the absence of reliable anatomic landmarks.

Imageless robotic assistance introduces several advantages in this context. Unlike conventional techniques, the workflow allows the knee to be treated as a native joint during the initial acquisition phase, despite the presence of a UKA implant. Hip center acquisition and full kinematic mapping are performed prior to explantation, enabling capture of global alignment and dynamic soft-tissue behavior before surgical alteration of the joint. However, the use of dual-stage imageless robotic-assisted TKA in conversion UKA is not well described in the literature [5].

The key originality of this workflow lies in this two-step strategy: first, native kinematic registration prior to implant removal; second, defect quantification through supplementary reference acquisition integrated into the robotic model after explantation. Additional femoral and tibial reference points allow precise delineation of bone defects and refinement of resection depth in real time. This iterative registration process facilitates conservative resections and may reduce the need for stems or augments. Bone preservation is particularly relevant in conversion surgery, as excessive resection during the first revision may compromise future reconstructive options. By combining kinematic mapping, quantitative defect assessment, and dynamic gap analysis, the imageless robotic workflow supports controlled and individualized reconstruction. Early data suggest that robotic-assisted conversion techniques do not appear inferior to conventional conversion techniques (CCT), although more robust clinical outcome data are required [6]. In a matched case–control study, Tuecking et al. reported that imageless robotic-assisted UKA-to-TKA revision resulted in limb alignment accuracy, resection depth, and insert selection comparable to primary robotic-assisted TKA, suggesting that robotic workflows may mitigate some of the technical disadvantages traditionally associated with conversion procedures [7].

While, for instance, robotic systems seem to have held some promise for better improvement in implant positioning and alignment, it is still quite unclear whether this development in technological advancement will translate into better long-term outcomes such as improved implant longevity and lower revision rates [8].

In the present case, use of the CORI™ system enabled detailed intraoperative planning, accurate bone resection, and real-time assessment of soft tissue balance. A great advantage of this workflow is the possibility to treat the knee as a native joint during the initial acquisition phase, despite the presence of a UKA implant. Hip center acquisition and full kinematic registration are performed prior to implant removal. This allows for the capture of the global limb alignment, functional deformity, and dynamic soft-tissue behavior in extension and flexion before surgical alteration of the joint.

Early kinematic mapping enables preservation of patient-specific alignment parameters and facilitates a functional planning strategy rather than a defect-driven reconstruction.

After implant removal, the CORI™ system allows acquisition of additional femoral and tibial reference points directly on the exposed bone surfaces. These supplemental points are integrated into the three-dimensional model and visually highlighted, enabling precise delineation of defect areas. This capability is particularly relevant in UKA conversion, where bone loss is often asymmetric and localized. This information influences resection depth, implant positioning, and the decision regarding stems or augments. Such defect quantification is difficult to reproduce with conventional instrumentation, where resection depth is generally determined relative to standard reference guides rather than individualized surface mapping. Bone preservation is particularly important in UKA-to-TKA conversion, as excessive resection at the first revision may compromise future reconstructive options. The ability to modulate resection depth in real time represents a meaningful advantage over conventional techniques.

This workflow may be particularly useful in cases with limited bone loss where preservation of host bone and avoidance of revision components are desirable.

By combining native kinematic mapping, quantitative defect assessment, and updated post-explant [9] registration, the robotic workflow facilitates conservative resections. In the present case, this strategy allowed restoration of alignment and symmetric flexion-extension gaps without stems or augments [10].

This report describes a single technical case and therefore does not permit conclusions regarding clinical outcomes, implant survivorship, or reproducibility across different patterns of bone loss [11]. Although early alignment accuracy was satisfactory and stems were avoided, further comparative studies are required to determine whether these technical advantages translate into improved clinical outcomes and implant survivorship.

## Conclusions

The robotic-assisted conversion of failed medial UKA to TKA using the CORI™ system is feasible and bone-conserving. The principal innovation of this technique is a dual-phase robotic registration strategy, consisting of pre-explant native kinematic registration followed by post-explant defect mapping and iterative intraoperative re-planning within a single imageless system. This workflow enables more precise characterization of bone loss, supports conservative bone resections, and facilitates accurate component positioning with controlled flexion–extension gap balancing. In selected cases, it may reduce the need for stems or augments.

Further comparative studies are required to determine whether these technical advantages translate into improved clinical outcomes and long-term implant survivorship.

## Data Availability

The video is available as supplementary material.

## Supplementary material

Supplementary *Video 1*: Revision of UKA to TKA using the CORI imageless system. The supplementary material of this article is available at https://www.sicot-j.org/10.1051/sicotj/2026045/olm.
